# COVID-19-Induced GI Vascular Abnormalities: Beyond Fatigue and Lungs

**DOI:** 10.7759/cureus.110476

**Published:** 2026-06-08

**Authors:** Marawan Zaki, Tom J Berriman, Eman Sultan, Summan Jannat

**Affiliations:** 1 Gastroenterology and Hepatology, York Hospital, York, GBR; 2 General Internal Medicine, York Hospital, York, GBR

**Keywords:** angioectasia, anigodysplasia, covid-19, gastro-intestinal bleed, iron deficiency anemia (ida)

## Abstract

COVID-19 infection is associated with multi-system complications other than primary pulmonary disease. It is well-documented that it causes an inflammatory response, which in severe cases can lead to pathologies in other organ systems. Gastrointestinal (GI) manifestations are common and includes anorexia, diarrhoea, nausea, vomiting, and abdominal pain. An uncommon GI manifestation in COVID-19 infection is GI bleeding (GIB). Angioectasia is one of the rare causes of GIB, not very common, reasonably straightforward to diagnose, but difficult to treat, with no consensus in the literature on the most effective treatment modalities and no standardised approaches to management. In this paper, we present an unusual case of GIB following a severe COVID-19 infection, discussing the diagnostic and therapeutic challenges. Written and informed consent to publish was obtained from the patient

## Introduction

A literature review reported that common gastrointestinal (GI) manifestations of COVID-19 included anorexia, diarrhea, nausea, vomiting, and abdominal pain. The pooled prevalence of these is estimated to be 17.6% from an early meta-analysis [1]. GI bleeding (GIB) is an unusual manifestation with a reported prevalence of 2.1% in a recent meta-analysis [2]. Angioectasia is one of the rarer causes of GIB and a recent multicentre cross-sectional study focusing on patients with COVID-19 infection showed a prevalence of 1.2 and 7.4% for upper and lower GI tracts, respectively [3], which is higher than the 0.092% prevalence found in a recent large population-based retrospective study [4]. Of note, there are no long-term studies on COVID-19 and gastrointestinal outcomes. This could indicate an unstudied disease burden similar to long Covid syndromes.

Angioectasias are defined as small, tortuous, ectatic vessels seen in the mucosal and submucosal layers of the bowel. They are the most common vascular anomaly encountered in the gastrointestinal tract [5]. Pathogenesis remains poorly understood; there are however some known associations: aortic stenosis, von Willebrand disease (hereditary or acquired), chronic kidney disease (CKD) [5], and left ventricular assist devices [6].

## Case presentation

A 64-year-old lady presented to our care after collapsing at home with sharp intrascapular pain with no associated loss of consciousness. She has a background history of hiatus hernia, gout, and obstructive sleep apnoea (OSA) managed with home continuous positive airway pressure (CPAP) therapy and a family history of ovarian cancer. On initial assessment, she was diagnosed with a traumatic left fibular fracture and tested positive for COVID-19 as part of routine admission screening. She had no signs of respiratory distress initially and of note has no murmurs on cardiovascular examination.

After one week of hospital stay and while investigating the cause of collapse, she developed significant hypoxia requiring high flow nasal oxygen (HFNO2) and continuous CPAP therapy, necessitating transfer to the intensive care unit (ICU) for respiratory support. Repeat blood work showed normal white cell count with increase in C-reactive protein to 63. Further chest imaging showed left basal atelectasis and consolidation. She was treated as severe COVID-19 pulmonary infection with superadded bacterial infection. Treatment followed local protocols and was given intravenous dexamethasone with oral proton pump inhibitor cover, remdesivir and intravenous antibiotics. Prophylactic subcutaneous enoxaparin was started on admission and then switched to subcutaneous dalteparin on starting treatment for severe COVID-19 infection.

On admission, blood tests showed an iron deficiency anaemia (IDA) (see Table 1). She was treated with packed red blood cells (PRBC) transfusions, which improved her hemoglobin (Hb). Two weeks into the admission, she had an episode of melena with a significant drop in Hb, requiring further PRBC transfusions. Computed tomography(CT) imaging of the thorax, abdomen and pelvis as part of the IDA assessment revealed an ovarian cyst with no radiological evidence of other masses or features suggestive of malignancy. Subsequently, she went on to have an inpatient gastroscopy that showed moderate non-erosive gastritis, which was felt insufficient to explain the anaemia. Colonoscopy was not done at the time, as the patient was not well enough to tolerate this. She improved from her COVID-19 infection, no longer requiring respiratory support, and stepped down from ICU after five days with no further episodes of melena with stable Hb. She remained admitted for a total of 18 days and was discharged with a plan for further outpatient endoscopy and gynaecology follow-up. The ovarian cyst was deemed benign after repeat imaging and a period of surveillance.

**Table 1:** Summary of patient laboratory data at admission Hb = Haemoglobin, MCV = Mean corpuscular volume, MCH = Mean corpuscular haemoglobin

	Admission	Normal range
Hb	60	115-165 g/L
MCV	83	80-100 FL
MCH	25.0	27.0-32.0 pg
Folate	7.4	>3.9 ug/L
B12	379	197-771 ng/L
Ferritin	6	30-260 ug/L
Hematocrit	0.20	0.360-0.470 L/L

Two months after discharge, follow-up bloods showed stable Hb. She was seen in the gastroenterology clinic one year later; bloods now showed recurrence of IDA (see Table 2), and the patient was now reporting rectal bleeding. Following this visit, she had both gastroscopy and colonoscopy, which were reported as normal. She was started on oral iron replacement therapy and was referred for capsule endoscopy.

**Table 2:** Summary of patient laboratory data 11 months post-admission Hb = Haemoglobin, MCV = Mean corpuscular volume, MCH = Mean corpuscular haemoglobin

	11 months post-admission	Normal range
Hb	55	115-165 g/L
MCV	80	80-100 FL
MCH	23.1	27.0-32.0 pg
Folate	5.2	>3.9 ug/L
B12	338	197-771 ng/L
Ferritin	6	30-260 ug/L
Hematocrit	0.19	0.360-0.470 L/L

**Figure 1:**
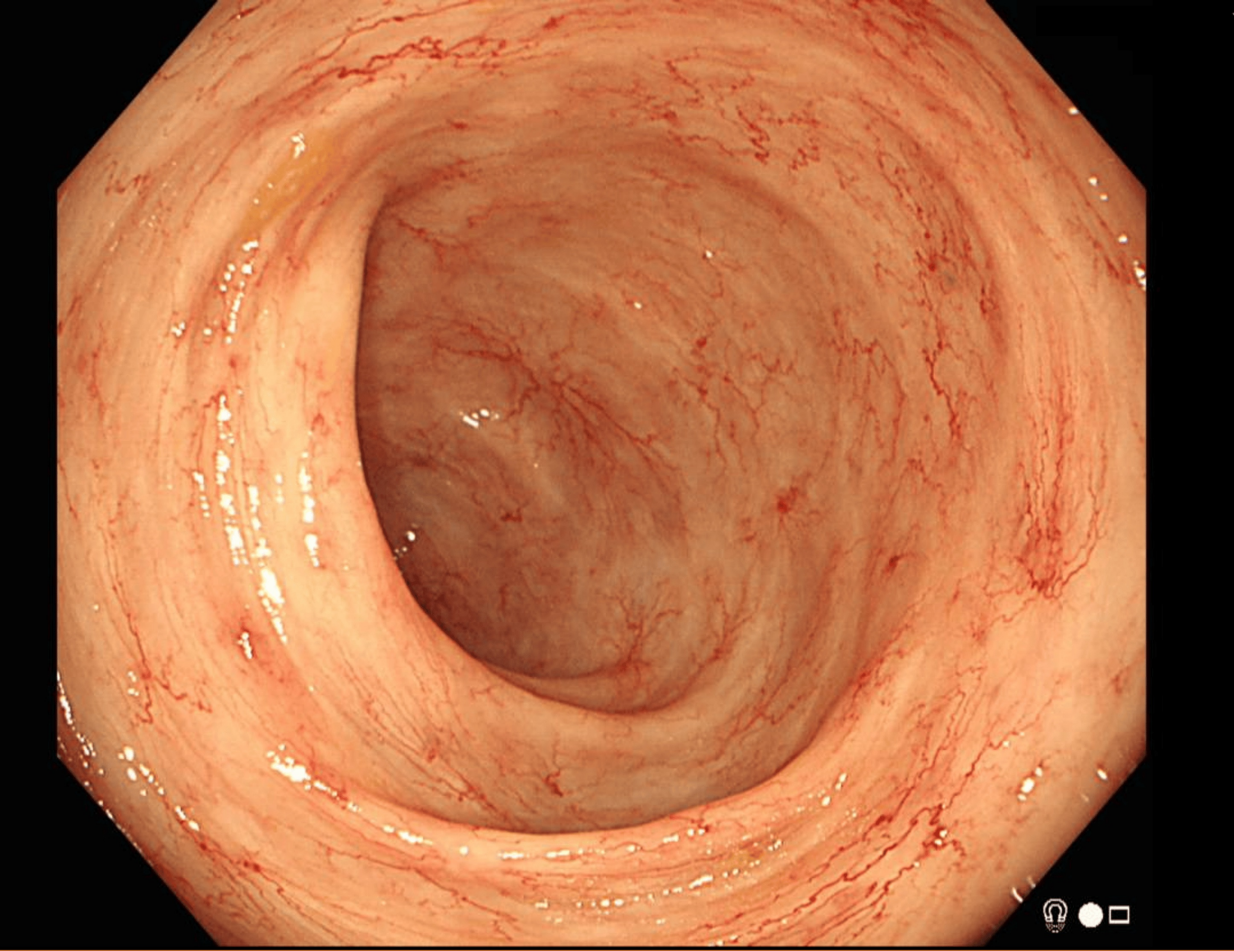
Colonoscopy: 12 months post initial admission Widespread colonic angiodysplasia

During the following five months, the rectal bleeding recurred and had two further presentations to hospital with anaemia requiring both PRBC transfusions and intravenous iron supplementation. At 12 months after her first presentation, an urgent repeat colonoscopy was requested and showed diffuse prominent capillaries and telangiectasias throughout the colon (Figure 1). This was too widespread for endoscopic treatment. Her capsule endoscopy was prioritised to the earliest availability and was subsequently unremarkable with one single small bowel telangiectasia. Reviewing her current blood work and compared to previous results, she has a stable estimated glomerular filtration rate (eGFR) of 50-55, consistent with CKD stage 3. She was also referred to haematology to rule out acquired von Willebrand disease due to well-documented associations with GI telangiectasia and was ruled out.

The ongoing IDA and endoscopic findings were then discussed with the patient. Endoscopic treatment was not suitable due to the widespread distribution of the telangiectasia; the options were therefore octreotide or thalidomide. After discussing the evidence for these, side effects and route of administration, she preferred thalidomide and was started on 50 mg twice a day for four months. She was given a patient information leaflet for the side effects (e.g. sedation, constipation, peripheral neuropathy and embolism) and safety netting for when to seek urgent medical help with ongoing pharmacist follow-up to monitor for any suggestion of these.

Her symptoms resolved, but she had a relapse of rectal bleeding and IDA 24 months after this course. She was treated with a second three-month course of thalidomide at the same dosage. At 18 months, post-second course of treatment, there has been no further rectal bleeding with normalisation of her haemoglobin and iron studies (see Table 3).

**Table 3:** Summary of patient laboratory data post thalidomide treatment Hb = Haemoglobin, MCV = Mean corpuscular volume, MCH = Mean corpuscular haemoglobin

	Post 1st Thalidomide course	24 months Post 1st Thalidomide course	Post 2nd Thalidomide course	Normal range
Hb	137	88	131	115-165 g/L
MCV	95	77	95	80-100 FL
MCH	34.3	22.4	32.8	27.0-32.0 pg
Folate	6.8	10.0	6.6	>3.9 ug/L
B12	295	344	380	197-771 ng/L
Ferritin	112	13	55	30-260 ug/L
Hematocrit	0.38	0.30	4.00	0.360-0.470 L/L

## Discussion

Several studies since the pandemic have explored GIB with COVID-19. A prospective study focusing on COVID-19 patients found nearly half had significant acute GI mucosal injury [7]. Two recent systematic reviews suggest a GIB prevalence in hospitalised patients between 1.5 - 3% [8,9]. The available data suggests nearly 60% of inpatients are managed conservatively while those who undergo endoscopy most commonly show peptic ulcer disease and ischaemic-like colopathies [3,10]. In our literature search, only one multi-centre study [2] has described a finding of GI angioectasia, with none describing their development several months after an infection.

Pathogenesis of angioectasia remains to be fully understood. The known associations of angioectasia with aortic stenosis, von Willebrand disease and CKD suggest some possible mechanisms for the development of these aberrant vessels, such as mucosal and submucosal ischaemia/hypoxia, vascular endothelial growth factor (VEGF)-dependent angiogenesis and decreased platelet adhesion, respectively [5]. There are a number of factors in this patient that could contribute to the development of angioectasia including age, CKD and potentially chronic hypoxia related to OSA. COVID-19 infection also leads to endothelial dysfunction and the concomitant hypoxia that occurs with a pulmonary infection may potentially play a role [11].

This should also be viewed with some caution, as the known associations of aortic stenosis, von Willebrand disease, CKD and treatment of COVID-19 with dexamethasone and anticoagulants can also lead to the conclusion that these lead to increase risk of bleeding from angioectasia rather than necessarily causing their development.

There is an abundance of literature looking at acute and long-term manifestations of COVID-19, but none appeared in our search with a focus on GI pathology. This represents a potential new area of research with an unknown disease burden alongside further research into understanding the physiological mechanisms of angioectasia development.

Treatment is with either octreotide [12] or thalidomide [13]. A randomised controlled trial suggests a stronger effect of thalidomide in reducing bleeding episodes at doses of either 50 mg or 100 mg daily [13]. The choice of either agent is mainly guided by the patient's choice of route of administration and acceptability of side effects.

## Conclusions

In conclusion, GI symptoms are a common manifestation of COVID-19 infections, with GIB representing a small but significant percentage of these. Currently, there is a lack of studies that evaluate long-term GI complications after COVID-19 infection, especially GIB. There is likely to be an unknown disease burden, and further research is required to assess and explore this.
